# Maternal Adverse Childhood Experiences and Biological Aging During Pregnancy and in Newborns

**DOI:** 10.1001/jamanetworkopen.2024.27063

**Published:** 2024-08-09

**Authors:** Christian K. Dye, Daniel M. Alschuler, Haotian Wu, Cristiane Duarte, Catherine Monk, Daniel W. Belsky, Seonjoo Lee, Kieran O’Donnell, Andrea A. Baccarelli, Pamela Scorza

**Affiliations:** 1 Department of Environmental Health Sciences, Columbia University Mailman School of Public Health, New York, New York; 2 Department of Psychiatry, Columbia University, New York, New York; 3 Division of Behavioral Medicine, New York State Psychiatric Institute, New York; 4 Department of Obstetrics and Gynecology, Columbia University, New York, New York; 5 Department of Epidemiology, Butler Columbia Aging Center, Columbia University Mailman School of Public Health, New York, New York; 6 Department of Biostatistics, Columbia University Mailman School of Public Health, New York, New York; 7 Yale Child Study Center, Yale School of Medicine, New Haven, Connecticut

## Abstract

**Importance:**

Adverse childhood experiences (ACEs), potentially traumatic experiences occurring before the age of 18 years, are associated with epigenetic aging later in life and may be transmitted across generations.

**Objective:**

To test evidence of the transmission of biological embedding of life experience across generations by analyzing maternal ACEs and epigenetic clocks measured in mothers during pregnancy and in their children at birth.

**Design, Setting, and Participants:**

For this cross-sectional study, data from the Accessible Resource for Integrated Epigenomic Studies (ARIES) substudy of the Avon Longitudinal Study of Parents and Children (ALSPAC) were analyzed. The ALSPAC study recruited 14 541 women who gave birth in the Avon Health District in the UK between April 1, 1991, and December 31, 1992. The ARIES substudy comprised 1018 mother-offspring dyads based on the availability of DNA samples profiled in 2014. Epigenetic age was estimated using DNA methylation-based epigenetic clocks (including Horvath, Hannum, GrimAge, PhenoAge, and DunedinPACE) in mothers during pregnancy and the Knight and Bohlin cord blood epigenetic clocks in newborns. Analyses were performed between October 1, 2022, and November 30, 2023.

**Exposures:**

A composite measure of maternal ACEs was the primary exposure in both maternal and offspring models; as a secondary analysis, individual ACEs were measured separately. The Edinburgh Postnatal Depression Scale (EPDS) was used to investigate depression during pregnancy as an exposure.

**Main Outcomes and Measures:**

Changes in epigenetic age acceleration (EAA) were investigated as the primary outcome in maternal models during pregnancy. Changes in epigenetic gestational age acceleration (GAA) were the primary outcome in offspring analyses. Linear regression analyses were used to determine the association between maternal ACEs and both outcomes.

**Results:**

This study included 883 mother-child dyads. The mean (SD) maternal age at delivery was 29.8 (4.3) years. Pregnant women with higher ACE scores exhibited higher GrimAge EAA (β, 0.22 [95% CI, 0.12 to 0.33] years; *P* < .001). Maternal ACEs were not associated with GAA in newborns using *P* < .05 as a cutoff to determine statistical significance. Depression was associated with higher GrimAge EAA (β, 0.06 [95% CI, 0.02 to 0.10] years; *P* = .01) in mothers during pregnancy, but not in newborns, and did not mediate the association between ACEs and EAA.

**Conclusions and Relevance:**

The findings of this study suggest that maternal ACEs may be associated with epigenetic aging later in life, including during pregnancy, supporting a role for maternal ACEs in offspring development and health later in life.

## Key Points

**Question:**

Are maternal adverse childhood experiences (ACEs) associated with epigenetic aging during pregnancy and transmitted across generations?

**Findings:**

In this cross-sectional study of 883 mother-child dyads, pregnant women with higher composite ACE scores were epigenetically older.

**Meaning:**

The findings of this study suggest that maternal ACEs may become biologically embedded and may contribute to understanding the association of ACEs with health later in life and intergenerationally.

## Teaser

This cross-sectional study uses DNA methylation-based epigenetic clocks to assess the association between maternal adverse childhood experiences and biological aging during pregnancy and in newborns.

## Introduction

Adverse childhood experiences (ACEs) are potentially traumatic experiences that occur during childhood and adolescence and have been associated with premature morbidity and mortality.^1^ In the US, 45% of children have experienced at least 1 ACE, and 10% have experienced at least 3 ACEs.^2^ Adverse childhood experiences are associated with an increased risk of adverse mental and physical health during pregnancy as well as detriments to offspring development, behavior, and health.^3,4,5,6,7,8,9^ Indeed, evidence suggests that accumulation of ACEs is associated with increased depression later in life and poorer health outcomes in both mothers (including age-related diseases such as cardiovascular diseases, type 2 diabetes, cancer, and dementia) and newborns (including overall health-related quality of life, which has been associated with age-related disease risk).^10,11,12,13^ Although the association between maternal ACEs and offspring health is established and behavioral mechanisms are proposed, less is known regarding the underlying biological mechanisms involved.

Epigenetic mechanisms, including DNA methylation, may underlie the association of ACE exposure with later health and the next generation.^14,15^ DNA methylation patterns have been used as biomarkers of biological aging,^16^ the process by which the integrity and resilience capacity of cells, tissues, and organs decrease over the lifespan.^17,18^ DNA methylation-based biomarkers of aging, or epigenetic clocks, have been associated with a spectrum of conditions, including cardiovascular diseases, type 2 diabetes, and cancer.^19,20^ Multiple studies have reported that ACEs are associated with advanced epigenetic aging.^21,22,23,24,25,26^ In previous studies, investigators found that maternal ACEs were associated with epigenome-wide changes in DNA methylation in newborn cord blood^27,28^ and in epigenetic aging changes in children.^29^ Evidence suggests that altered epigenetic aging can be programmed early in development,^30^ even in utero, increasing disease risk over a person’s lifespan.^31^ Furthermore, ACEs may have lasting effects on mental health through adulthood, including depression,^13,32,33^ which is associated with epigenetic aging changes.^34^ Depression during pregnancy has been associated with adverse newborn health, which is associated with advanced epigenetic aging, suggesting that depression has a role in ACE-associated epigenetic aging in mothers and newborns.^35^ Although epigenetic clocks have been useful to understand biological aging associated with ACEs, their examination across multiple generations, including during pregnancy and in newborns, has yet to be fulfilled.

In this study, we examined whether ACEs of mothers were associated with heightened epigenetic age acceleration (EAA) during pregnancy and epigenetic gestational age acceleration (GAA) in their newborns. We also investigated whether depression mediated these associations.

## Methods

### Study Population

This cross-sectional study used data from the Accessible Resource for Integrated Epigenomic Studies (ARIES), a substudy of the Avon Longitudinal Study of Parents and Children (ALSPAC). Written informed consent was obtained from mothers, and mothers gave consent for participating children in the ALSPAC and ARIES studies. Ethical approval was obtained from the ALSPAC Ethics and Law Committee and the local research ethics committees. Consent for biological samples was collected in accordance with the UK Human Tissue Act 2004. Informed consent for the use of data collected via questionnaires and clinics was obtained from participants following the recommendations of the ALSPAC Ethics and Law Committee at the time. This study was preregistered with the Open Science Framework^36^ and followed the Strengthening the Reporting of Observational Studies in Epidemiology (STROBE) reporting guideline.

The ALSPAC investigators recruited 14 541 women who gave birth in the Avon Health District in the UK between April 1, 1991, and December 31, 1992,^37,38^ out of 20 248 pregnancies identified as being eligible. Of the initial pregnancies, there were 14 676 fetuses, resulting in 14 062 live births and 13 988 children who were alive at age 1 year. The participation rate was between 85% and 90% and included families reflecting the general UK population, although less ethnic minority representation was unintentionally introduced (3% in ALSPAC vs 7.6% in the UK). Initiated in 2012 as a substudy to ALSPAC, ARIES included a subset of 1018 mother-child ALSPAC dyads recruited during pregnancy. Mothers in the ARIES study were slightly older, were more likely to have a nonmanual occupation, and were less likely to have smoked throughout pregnancy compared with the overall ALSPAC sample population. In the ARIES study, DNA was purified from peripheral blood samples from mothers during an antenatal visit, and DNA for newborns was from umbilical cord blood at birth. Quantification of DNA methylation was performed during 2014 and made available to investigators thereafter. For this analysis, we included 883 of the 1018 mother-child dyads (eFigure 1 in Supplement 1). The ALSPAC website^39^ contains details of all of the data that are available through a fully searchable data dictionary and variable search tool.

### Data Collection

Enrolled mothers submitted retrospective self-reports of ACEs at 18 to 32 weeks of gestation and when their child was aged approximately 3 years through mail-in questionnaires.^40^ For consistency with the literature, we analyzed a cumulative measure of 10 ACEs,^41^ including emotional abuse, physical abuse, sexual abuse, emotional neglect, physical neglect, loss of a parent due to separation or death, domestic violence, family member with addiction, family member mental illness, and family member incarceration. Questionnaires were coded (yes or no) for each ACE variable. A sum was created as a cumulative ACE score ranging from 0 to 10.

Maternal age was self-reported during gestational week 8. Parity and maternal smoking during pregnancy, calculated as the number of cigarettes smoked per day during the first trimester, were reported at around 18 weeks of gestation. Maternal education was determined by self-report at around 32 weeks as less than high school, high school but no further education, some college or technical training, or completed college. Infant gestational age at birth was taken from obstetrics records determined by last menstrual period. Prepregnancy body mass index (BMI; calculated as weight in kilograms divided by height in meters squared) was calculated from self-reports at 12 weeks of gestation. Approximately 44% of prenatal maternal samples and 82% of cord blood samples were white blood cells, whereas the remaining samples were either whole blood (approximately 56% of prenatal samples) or blood spots collected from umbilical cord blood (18% of cord blood samples). Because of sample-type heterogeneity, we included sample type as a covariate in statistical analyses.

Given that depression is increased in individuals with higher exposure to ACEs and depression has been associated with advanced epigenetic aging, we examined depression as a potential mediator of ACEs on changes in epigenetic aging and health in mothers and newborns. We used the Edinburgh Postnatal Depression Scale (EPDS) to assess depression symptoms in pregnant women at 18 and 32 weeks of gestation, with a 10-question survey scored across each question; scores were then summed to create a total summary score.^42^ An EPDS score greater than 10 reflects a probable case of major depression in pregnant women.^43^

### DNA Methylation Preprocessing, Normalization, and Quantification

DNA methylation was measured using peripheral blood samples collected from 1018 mothers during antenatal clinic visits and from umbilical cord blood samples taken at birth for newborns, detailed elsewhere.^44,45^ DNA samples from maternal peripheral blood were collected at a mean (SD) gestational age of 25.7 (9.5) weeks. DNA was bisulfite converted using the EZ DNA Methylation Kit (Zymo Research), and DNA methylation was quantified using the Infinium Human Methylation 450K BeadChip array (Illumina Inc).

Initial quality control was performed using GenomeStudio, version 2011.1 (Illumina Inc). Raw IDAT files were preprocessed and normalized using the meffil package.^46^ Samples whose average probe intensities were unreliable (detection *P* ≥ .01), failing initial quality control, were repeated on the assay and were excluded if still unsuccessful. Samples in which genotype information did not match with single-nucleotide variation data were excluded. For those samples that lacked genome-wide single-nucleotide variation data, we used algorithms to estimate sex, and samples with discordant estimated sex and reported sex were removed. Failed CpG probes (detection *P* ≥ .01 in ≥5% of participants) and sex chromosome CpGs were removed. The data were then normalized via functional normalization, a between-array normalization method that removes technical variation in DNA methylation by using internal control probes.^47^ Chip effects were regressed on the raw β values before normalization and on the control matrix. Each sample cell-type composition was measured with the “estimateCellCounts” function in the minfi package using the Reinius references panel for peripheral blood in mothers and the Bakulski panel for newborn cord blood (monocytes, natural killer cells, B cells, CD4^+^ T cells, CD8^+^ T cells, and granulocytes, in addition to nucleated red blood cells in newborn cord blood).^48,49,50^

### Epigenetic Clock Calculation

We measured maternal biological aging using 5 DNA methylation clocks, including 2 first-generation epigenetic clocks developed to estimate chronological age (Hannum clock^51^ and the pan-tissue Horvath clock^52^), 2 second-generation clocks developed to estimate risk of death (GrimAge^53^ and PhenoAge^54^), and the pace-of-aging clock DunedinPACE,^24^ developed to estimate the rate of multiple organ systems deterioration. The Hannum, Horvath, PhenoAge, and GrimAge clocks were calculated using the PCmethod developed by Higgins-Chen et al^55^ to improve their reliability of measurement using the PC-clock code in the R environment, version 4.1 (R Project for Statistical Computing).^56^ DunedinPACE was calculated using the “PACEprojector” function in the DunedinPACE package in R.^57^

We measured newborn epigenetic gestational age from cord blood samples collected at birth using the epigenetic clocks described in Bohlin et al^58^ and Knight et al.^59^ These clocks were developed to estimate epigenetic gestational age at delivery from fetal umbilical cord blood. To calculate Bohlin- and Knight-derived gestational age, we used the methylclock package in R.

For all analyses, we regressed clock values on chronological age and estimated epigenetic age, and we obtained estimated residual values, commonly referred to as *age acceleration residuals*. For consistency with previous literature,^24^ we refer to positive residual values as indicating higher EAA and negative residual values as indicating lower EAA. Because DunedinPACE represents the pace of aging, values estimated by DunedinPACE were directly used for downstream analyses.

### Statistical Analysis

As the primary analysis, we used linear regression models with total ACE score as the primary estimator and EAA (ie, residuals between chronological age and epigenetic age) as the outcome, testing each clock separately. Prior evidence suggests that maternal ACEs may differentially affect behavioral patterns, health-related quality of life, psychological health, and physiologic differences between male and female individuals.^9,60,61,62^ Therefore, analyses for cord blood were stratified by infant sex. For maternal analyses, our primary model adjusted for a priori–selected covariates, including maternal age during pregnancy, parity, mean number of cigarettes smoked daily during the first trimester, maternal education, prenatal BMI, maternal sample type (whole blood or white blood cells), and sample cell-type composition (CD4^+^ T cells, CD8^+^ T cells, natural killer cells, monocytes, B cells, and neutrophils). In newborn analyses, our primary model adjusted for the following: maternal age during pregnancy, parity, mean number of cigarettes smoked daily during the first trimester, maternal education, prenatal BMI, gestational age at birth, and newborn sample type (umbilical white blood cells or cord blood spot). We excluded newborn cell-type composition in our primary analyses (ie, primary model) because gestational clocks were trained on cord blood samples and controlling for cell-type composition had minimal effect on regressions between gestation age and clinical gestational age.^59^ However, because previous evidence suggests that the Knight and Bohlin clocks are associated with cell-type proportion,^63^ we included a sensitivity analysis in the offspring as a secondary model that included cord blood cell-type proportion as a covariate to confirm whether cell-type proportion affected effect size estimates. For maternal analyses, we included a sensitivity analysis as a secondary model in which we removed maternal cell-type composition as a covariate. As a subanalysis to our primary analysis, we tested each type of ACE separately as the primary estimator.

To explore whether depression was a mediator between ACEs and EAA in pregnancy and newborns, we used the EPDS summary score at 18 weeks of gestation to test maternal depression symptoms with biological age residuals in mothers and gestational age residuals in newborns. All models were analyzed similarly to our primary analyses between ACEs and changes in biological age residuals.

Data analysis was conducted with R, version 4.1 (R Project for Statistical Computing). Statistical significance was set at *P* < .05 (2-tailed). Analyses were performed between October 1, 2022, and November 30, 2023.

## Results

### Participant Characteristics

Of the 1018 mother-child dyads enrolled in ARIES, we retained 883 for this analysis. Table 1 presents a summary of participant characteristics. The mean (SD) maternal age at delivery was 29.8 (4.3) years. There were 389 male newborns (44.1%) and 398 female newborns (45.1%) (sex was missing for 96 [10.9%]). The mean gestational age of offspring at birth was 39.4 (1.6) weeks for male newborns and 39.7 (1.5) weeks for female newborns. The prorated mean (SD) total ACE score for mothers was 1.3 (1.5), and 93 women (10.5%) reported experiencing 3 or more ACEs. Sexual abuse was the most common ACE, identified by 256 participants (29.0%). The mean (SD) depression symptom score, as measured by the EPDS, was 6.3 (4.6), and 159 women (18.0%) had an EPDS score greater than 10. Most mothers (464 [52.6%]) were multiparous. A total of 106 mothers (12.0%) reported smoking during the first trimester of pregnancy and smoked a mean (SD) of 8.4 (7.2) cigarettes daily. The mean (SD) prepregnancy BMI was 22.7 (3.6). For education, 329 mothers (37.3%) had technical schooling or some college, and 301 (34.1%) had high school education.

**Table 1.** Descriptive Statistics of ALSPAC Mother-Child Dyads

Variable	All dyads (N = 883)^a^	Missing, No. (%) [range]
Maternal characteristic		
Age at delivery, mean (SD), y	29.8 (4.3)	NA [16.0-42.0]
Prepregnancy BMI, mean (SD)	22.7 (3.6)	45 (5.1) [14.2-45.2]
Parity (≥1 birth)	464 (52.6)	16 (1.8) [NA]
Smoking during pregnancy (yes)	106 (12.0)	<5 (<0.6) [NA]
No. of cigarettes smoked daily, mean (SD)	8.4 (7.2)	NA [1.0-30.0]
Education		
Less than high school	65 (7.4)	NA
High school or equivalent	301 (34.1)	NA
Technical school or some college	329 (37.3)	NA
College	188 (21.3)	NA
EPDS score, mean (SD)	6.3 (4.6)	27 (3.1) [0-25.0]
Newborn characteristic		
Sex		
Male	389 (44.1)	96 (10.9) [NA]
Female	398 (45.1)	
Gestational age at birth, mean (SD), wk		
Male	39.4 (1.6)	NA [30.0-43.0]
Female	39.7 (1.5)	NA [32.0-44.0]
Prorated maternal ACE score, mean (SD)^b^		
Total ACE score^c^	1.3 (1.5)	NA [0-8.0]
Emotional abuse	0.1 (0.3)	NA [0-1.0]
Physical abuse	0.04 (0.2)	10 (1.1) [0-1.0]
Sexual abuse	0.3 (0.5)	53 (6.0) [0-1.0]
Emotional neglect	<0.5 (<0.6)	1 (0.1) [0-1.0]
Physical neglect	<0.5 (<0.6)	1 (0.1) [0-1.0]
Parental death or separation	0.2 (0.4)	NA [0-1.0]
Parental domestic violence	0.1 (0.3)	55 (6.2) [0-1.0]
Parental substance use	0.1 (0.3)	NA [0-1.0]
Parental mental illness	0.3 (0.4)	NA [0-1.0]
Parental incarceration	0 (0.03)	NA [0-1.0]
Any type of family adversity^d^	0.4 (0.5)	21 (2.4) [0-1]
Any type of abuse^e^	0.3 (0.5)	53 (6.0) [0-1]
Any type of neglect^f^	<5 (<0.6)	2 (0.2) [0-1]

Abbreviations: ACE, adverse childhood experience; ALSPAC, Avon Longitudinal Study of Parents and Children; BMI, body mass index (calculated as weight in kilograms divided by height in meters squared); EPDS, Edinburgh Postnatal Depression Scale; NA, not applicable.

^a^ Unless stated otherwise, values are presented as No. (%) of participants.

^b^ Prorated for missing variables. Missing values for individual ACEs and categories of ACEs (any type) based on original subscore.

^c^ Cumulative (continuous) score culminating individual ACEs.

^d^ Presence of 1 or more of parental domestic violence, substance abuse, mental illness, death or separation, or incarceration.

^e^ Presence of 1 or more of physical, emotional, or sexual abuse.

^f^ Presence of 1 or more of physical neglect or emotional neglect.

Mothers, on average, exhibited higher epigenetic age relative to chronological age on all epigenetic clock estimates (eTable 1 in Supplement 1). For newborns, the mean (SD) epigenetic gestational age was within the typical range for both the Knight (38.8 [2.0] weeks) and Bohlin (39.7 [1.0] weeks) clocks (eTable 1 in Supplement 1). Overall, maternal epigenetic age derived from each epigenetic clock (Horvath, Hannum, PhenoAge, and GrimAge) was associated with maternal chronological age, although the pace-of-aging biomarker, DunedinPACE, was not associated with chronological age (eFigure 2A in Supplement 1). For newborns, gestational age was associated with epigenetic gestational age estimated using both the Bohlin and Knight clocks (eFigure 2B in Supplement 1).

### Maternal ACEs and EAA in Mothers

We observed an association between maternal total ACE scores and higher GrimAge EAA (β, 0.22 [95% CI, 0.12 to 0.33] years; *P* < .001; eTable 2 in Supplement 1) (Table 2). Maternal ACE scores were not associated with EAA derived from the PhenoAge, Horvath, Hannum, and DunedinPACE clocks. In our sensitivity analysis, removing cell-type composition did not diminish the association between maternal total ACE scores and GrimAge EAA (β, 0.22 [95% CI, 0.10 to 0.34]; *P* < .001).

**Table 2.** Association Between Maternal Total ACE Score and Epigenetic Aging During Pregnancy^a^

Epigenetic clock	β Coefficient (95% CI)	*P* value
Horvath	0.03 (−0.14 to 0.20)	.71
Hannum	0.04 (−0.10 to 0.18)	.57
PhenoAge	0.17 (−0.00 to 0.35)	.054
GrimAge	0.22 (0.12 to 0.33)	<.001
DunedinPACE	0.003 (0.00 to 0.01)	.21

Abbreviation: ACE, adverse childhood experience.

^a^ Total ACE score is based on a summed measure of 10 ACEs. Linear regression is adjusted for maternal sample type (white blood cell, whole blood), maternal age during pregnancy, parity, number of cigarettes smoked during the first trimester, maternal education, prenatal body mass index (calculated as weight in kilograms divided by height in meters squared), and maternal sample cell-type composition (CD4^+^ T cells, CD8^+^ T cells, natural killer cells, monocytes, B cells, and neutrophils).

**Figure 1.**
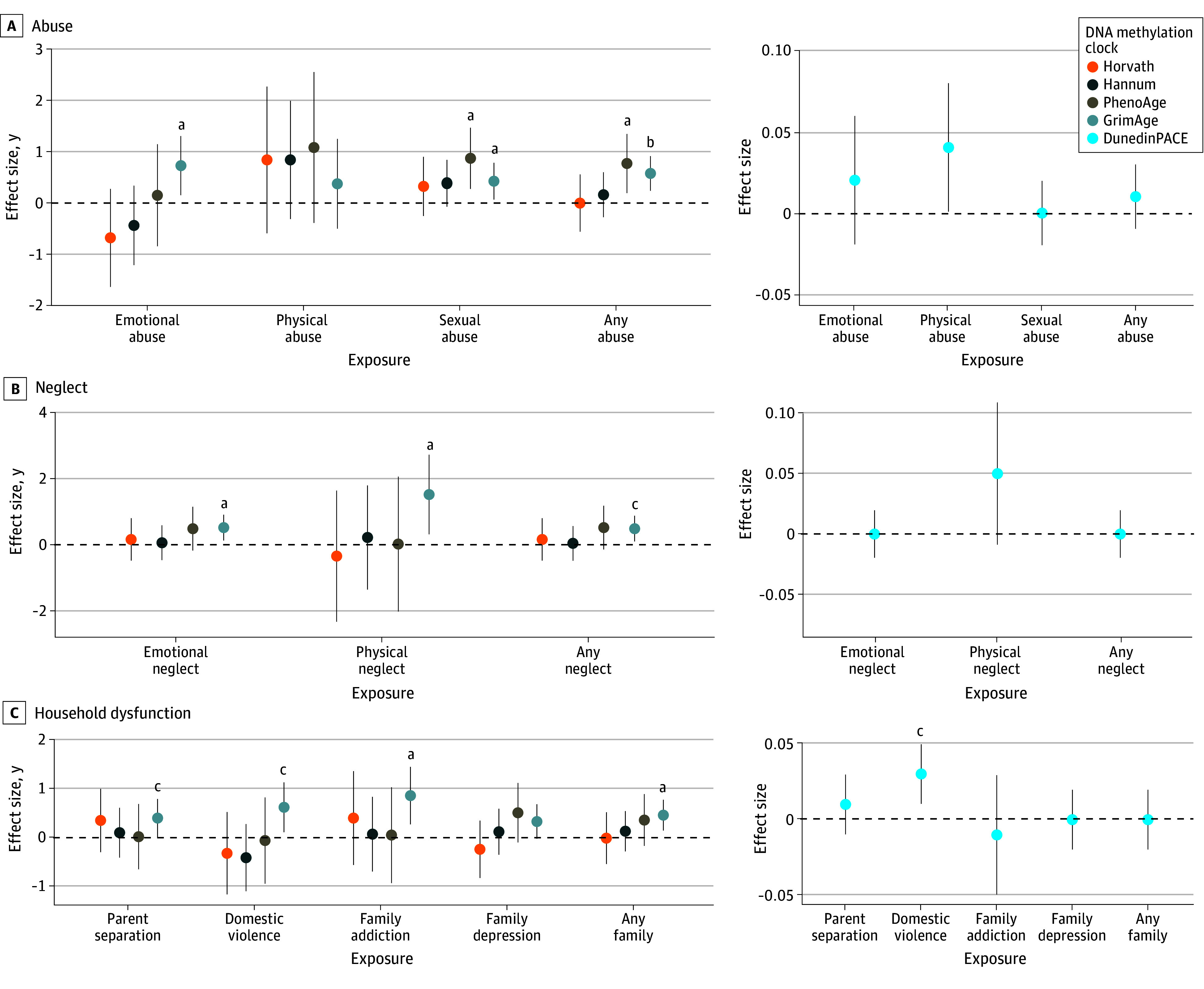
Association Between Maternal Adverse Childhood Experiences and Epigenetic Age Acceleration (EAA) in Pregnant Women Effect size estimates (in years [95% CIs]) for change in epigenetic age in prenatal blood from pregnant women, calculated using 5 DNA methylation clocks: Horvath, Hannum, PhenoAge, GrimAge, and DunedinPACE. Results are adjusted for maternal sample type (white blood cell, whole blood), maternal age during pregnancy, parity, number of cigarettes smoked during the first trimester, maternal education, prenatal body mass index (calculated as weight in kilograms divided by height in meters squared), and maternal sample cell-type composition (CD4^+^ T cells, CD8^+^ T cells, natural killer cells, B cells, monocytes, and neutrophils). ^a^*P* < .01. ^b^*P* < .001. ^c^*P* < .05.

Using individual ACE score components with mothers’ epigenetic clocks, we found that the following were associated with higher GrimAge EAA: any type of abuse, emotional abuse, and sexual abuse; any type of neglect, emotional neglect, and physical neglect; and any family adversities, parental separation or death, parental domestic violence, and parental addiction. Maternal exposure to any type of abuse and to sexual abuse was associated with higher PhenoAge EAA. Parental domestic violence was associated with a higher pace of aging according to DunedinPACE. The results are shown in Figure 1 and eTable 3 in Supplement 1.

### Maternal ACEs and Epigenetic GAA in Newborns

We conducted a sex-stratified analysis of maternal ACE associations with newborn differences in GAA (Table 3 and eTable 4 in Supplement 1). Maternal ACE score was not associated with GAA (eTable 4 in Supplement 1).

**Table 3.** Association Between Maternal Total ACE Score and Newborn Epigenetic Gestational Aging^a^

Epigenetic clock	β Coefficient (95% CI)	*P* value
Female		
Bohlin	0.01 (−0.04 to 0.07)	.60
Knight	−0.08 (−0.19 to 0.04)	.19
Male		
Bohlin	0.05 (−0.00 to 0.11)	.050
Knight	0.11 (−0.03 to 0.26)	.12

Abbreviation: ACE, adverse childhood experience.

^a^ Linear regression is adjusted for maternal age during pregnancy, parity, number of cigarettes smoked during the first trimester, maternal education, prenatal body mass index (calculated as weight in kilograms divided by height in meters squared), gestational age at birth, and newborn sample type (white blood cells, blood spots).

**Figure 2.**
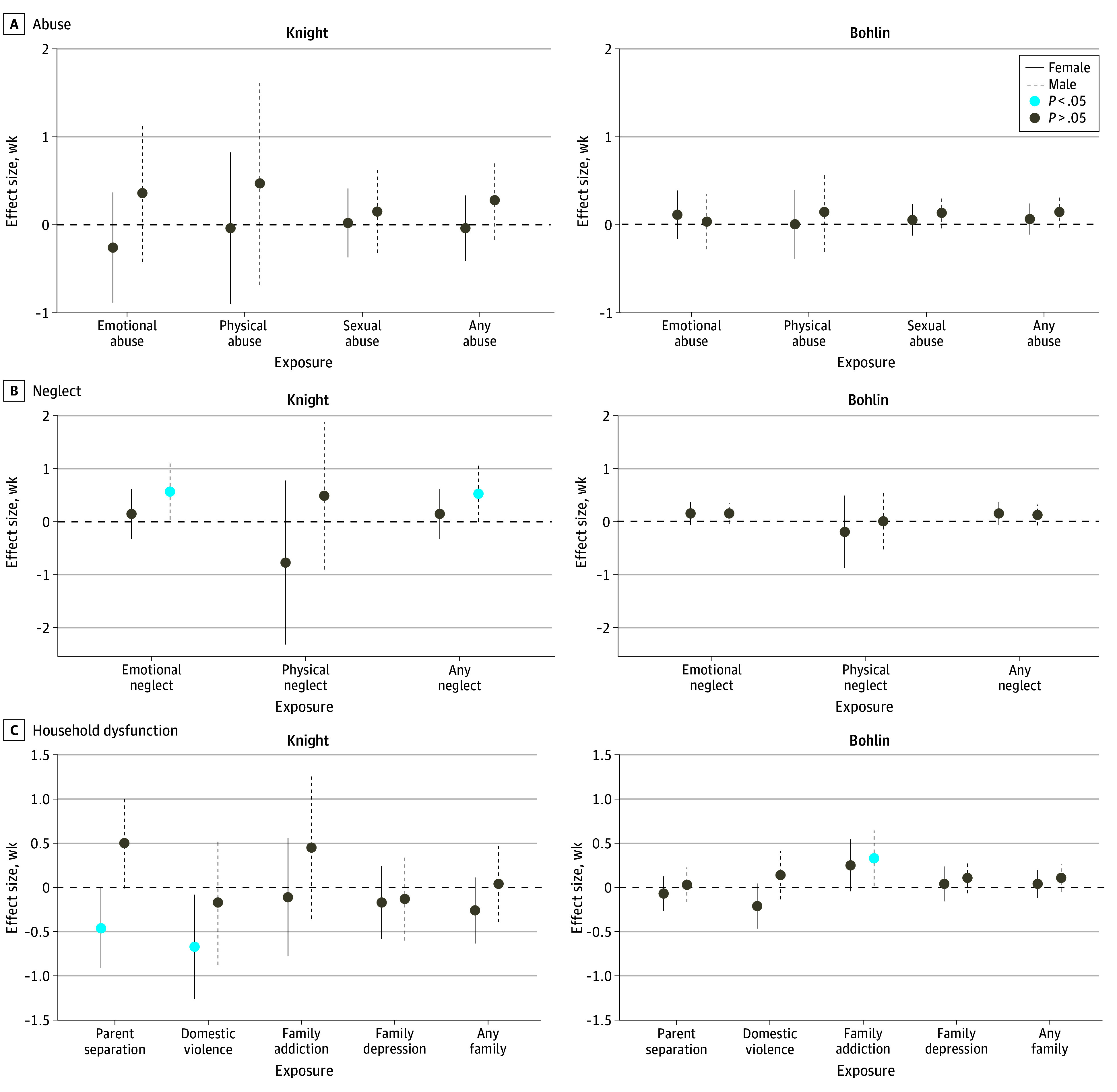
Association Between Maternal Adverse Childhood Experiences and Epigenetic Gestational Age Acceleration (GAA) in Newborns Effect sizes (in weeks [95% CIs]) for the change in epigenetic gestational age in cord blood collected from newborns, calculated using 2 clocks: Knight (left) and Bohlin (right). Results are adjusted for maternal age during pregnancy, parity, number of cigarettes smoked during the first trimester, maternal education, prenatal body mass index (calculated as weight in kilograms divided by height in meters squared), gestational age at birth, and newborn sample type (white blood cells, blood spot). Color schemes represent statistical significance (*P* < .05).

Next, we examined associations of individual maternal ACE score components with newborn epigenetic gestational clocks (Figure 2 and eTable 5 in Supplement 1). In male newborns, maternal exposure to any type of neglect and to emotional neglect was associated with higher Knight GAA. In female newborns, maternal exposure to parental separation or death and to parental domestic violence was associated with lower Knight GAA.

### Depression and EAA in Mothers and Newborns

 Edinburgh Postnatal Depression Scale score was positively associated with higher GrimAge EAA in mothers during pregnancy (β, 0.06 [95% CI, 0.02 to 0.10] years; *P* = .01) (eTable 6 in Supplement 1) but was not associated with GAA in either male or female offspring (eTable 7 in Supplement 1). Testing for mediation found no mediating role of maternal depression between maternal ACEs and epigenetic aging in mothers or in newborns (eTable 8 in Supplement 1).

## Discussion

In our analysis of mothers and offspring from the ARIES substudy of the ALSPAC, those who experienced more ACEs had higher GrimAge EAA during pregnancy compared with those with fewer ACEs. Overall, these results suggest the biological embedding of ACEs throughout the life course that is detectable during pregnancy. This finding is notable because biological aging is a potential indicator of negative health later in life^64^ and of health and development in offspring.^65^

Previous work in the ALSPAC identified that ACEs during childhood were associated with EAA through 17 years of age.^66^ Our study identified the association between ACEs and epigenetic aging in women later in life during pregnancy, a sensitive window for women and their developing offspring. In addition to a cumulative measure of ACE exposure, we found that mothers exposed to certain types of ACEs exhibited EAA during pregnancy. Our findings are consistent with prior results observing EAA later in life in those with higher cumulative ACE exposure.^26,66,67,68,69,70^ Differences in the association between ACEs and epigenetic aging during pregnancy were not affected by either the inclusion or omission of cell type. Given no differences by cell-type composition in our models, changes in epigenetic aging may be capturing the aging of tissues and organs rather than the gradual changes in immune cell composition that occur concurrently with the aging process.^71^ These results suggest that EAA during pregnancy may be an important indicator of physiologic processes of organ system integrity and functionality, which may contribute to both long-term disease susceptibility and the intergenerational effects of adverse exposures and aging during sensitive developmental windows, such as during pregnancy. Although research has found associations between ACEs and EAA,^26,72^ our findings are, to our knowledge, the first to show this association maintained during pregnancy.

Although our results for newborns were not significant at the threshold of *P* < .05 for determining statistical significance, given the move away from binary thinking regarding *P* values,^73^ our findings suggest that maternal ACEs may also affect the growing male neonate. This is consistent with previous findings of cumulative maternal ACEs being associated with increased biological aging in children and being more pronounced in male individuals.^29^ Further supporting these results, our group previously found that maternal ACEs are associated with genome-wide changes in DNA methylation in male newborns.^74^ This expands on previous observations of aging in childhood, in which these changes in epigenetic aging may become embedded as early as in newborns. Given that this study used cord blood samples obtained from newborns at birth, it was unlikely that changes in epigenetic aging were affected by postnatal parenting differences that have been shown to alter epigenetic aging patterns.^75^ Thus, adverse changes in epigenetic aging in newborns potentially occurred from prenatal adversities transmitted through maternal exposures that may be directly transmitted epigenetically and indirectly transmitted through biological and behavioral transmission that, in turn, have prenatal and postnatal epigenetic effects. Nonetheless, how changes in gestational aging affect development and health in newborns is not well understood, nor are any long-term health implications, and future work should seek to elucidate this association.

During fetal development, the neonate is affected by maternal environmental signals, potentially intended to increase fetal survival outside the womb, such as accelerated maturation,^76^ or to increase survival inside the womb, such as slow fetal growth so as to capitalize on available nutritional resources.^77^ Gestational epigenetic clocks have been valuable tools to understand both prenatal maternal conditions and newborn developmental outcomes,^78,79^ including evaluation of developmental maturity and birth weight (an indirect measure of maturity).^59,80,81^ This is notable because faster early childhood developmental trajectories can be detrimental to long-term health.^82,83^ Parental ACEs have also been associated with developmental delays in offspring,^84,85^ and lower gestational age and GAA have been associated with increased developmental delays.^86,87^ In preterm newborns, accelerated epigenetic aging was associated with impaired neurodevelopmental and behavioral development in newborns and early childhood.^65,88^ In both full-term and preterm newborns, higher GAA has been associated with adiposity^89^; in full-term babies, higher GAA is associated with greater birth weight, birth length, and head circumference, indicating heightened fetal development in males.^90^ Given that previous evidence found child sex may moderate the effects of maternal ACEs,^9^ the lower GAA in females observed in our study may be a sex-dependent effect on adaptation to maternal adversities during pregnancy.^91^ Our results suggest that maternal ACE exposure may have a different effect on male infants vs female infants, which may have important implications for developmental milestones and long-term health between sexes, although this needs to be further explored.

Conventionally, biological aging is a process by which the cells and tissues age. In newborns, this change in epigenetic gestational aging may not be capturing aging in the same way.^92^ Instead, the epigenetic age scores may be capturing epigenetic fetal reprogramming of organ and tissue developmental trajectories.^93^ In either case, these epigenetic changes that are captured by epigenetic aging occur in response to adverse environmental exposures, such as through the indirect effects of ACEs on physical health and psychosocial factors that may persist into pregnancy and intergenerationally.^94^ These changes in newborn epigenetic age that may be capturing epigenetic fetal reprogramming may be important to understanding development and health in postnatal life, including long-term consequences to health.^77,95^ Our results changed after adjusting for cell-type composition, suggesting that immune cell-type composition acts as an intermediary between maternal ACEs and gestational epigenetic aging. There is prior evidence that prenatal environmental exposures may contribute to the developmental programming of the immune system, which may lead to the expansion of immune cell subtypes that, although protective in the fetal environment, may be deleterious to health in the postnatal environment.^96,97^ Taken together, our findings indicate that maternal ACEs during pregnancy may have epigenetic effects on the developing fetus, thus underlying organ and immune cell developmental trajectories with implications for development and long-term health outcomes later in life.

### Limitations

Although this study provides compelling evidence, the results should be interpreted in the context of several limitations. The prevalence of ACEs in the ALSPAC sample was relatively low, although this is the case for much existing literature on the topic. Studies in samples exposed to relatively low levels of adversity might fail to detect associations, particularly those with small effect sizes as is the case with psychosocial exposures and intergenerational outcomes. Because our analysis was not performed in a population highly exposed to adversities, our findings might not generalize to such populations. Only 3% of ALSPAC mothers self-identified as belonging to a racial or ethnic minority group. Unfortunately, Black and Latinx populations sustain higher exposure to and experience more ACEs than their White counterparts.^98^ Future research should focus on populations with high exposure to adversity. Finally, maternal ACEs were self-reported by mothers through mail-in questionnaires and were thus likely susceptible to misclassification, such as reluctance to report sensitive ACEs (ie, social desirability bias). However, this misclassification is at least partially mitigated by the use of mail-in self-reports administered by the ALSPAC investigators to maintain participant confidentiality; in cases where it could have occurred, it is likely nondifferential given that our outcomes are based on molecular data that would not easily be observed.

## Conclusions

In this cross-sectional study, we found that the accumulation of adverse childhood experiences was associated with higher EAA in pregnant women. To our knowledge, this is the first study to test for differences in epigenetic aging in both pregnant women exposed to early life childhood adversities as well as in their newborns. Future studies should confirm these findings in more diverse cohorts, elucidate the long-term health effects of intergenerationally transmitted biological aging in the offspring, and further explore the mediating mechanisms between maternal adversity during childhood and changes in biological aging in offspring.

## Notes

Supplement 1. **eFigure 1.** Flowchart for Inclusion Criteria for Study **eTable 1.** Epigenetic Clock Statistics From Mothers and Newborns in ALSPAC **eFigure 2.** Correlation Matrix of Estimated Epigenetic Age and Chronological Age **eTable 2.** Association Between Maternal Epigenetic Aging During Pregnancy and Total Adverse Childhood Experience Score **eTable 3.** Association Between Individual Maternal Adverse Childhood Experience (ACEs) and Epigenetic Aging During Pregnancy **eTable 4.** Association Between Maternal Total Adverse Childhood Experiences (ACE) Score and Newborn Epigenetic Gestational Aging **eTable 5.** Association Between Individual Maternal Adverse Childhood Experiences (ACEs) and Newborn Epigenetic Gestational Aging **eTable 6.** Association Between Maternal Depression and Epigenetic Aging During Pregnancy **eTable 7.** Association Between Maternal Depression During Pregnancy and Newborn Epigenetic Gestational Aging **eTable 8.** Proportion Mediated by Maternal Depression During Pregnancy for the Association Between Maternal Total Adverse Childhood Experience (ACE) Score and Epigenetic Aging in Mothers and Newborns

Supplement 2.
Data Sharing Statement
