# Supplementary material for: Maternal Adverse Childhood Experiences and Biological Aging During Pregnancy and in Newborns
Source: JAMA Netw Open. 2024 Aug 9;7(8):e2427063. doi: 10.1001/jamanetworkopen.2024.27063 (PMC11316241; doi:10.1001/jamanetworkopen.2024.27063)
Supplement: Supplement 1. — eFigure 1. Flowchart for Inclusion Criteria for Study eTable 1. Epigenetic Clock Statistics From Mothers and Newborns in ALSPAC eFigure 2. Correlation Matrix of Estimated Epigenetic Age and Chronological Age eTable 2. Association Between Maternal Epigenetic Aging During Pregnancy and Total Adverse Childhood Experience Score eTable 3. Association Between Individual Maternal Adverse Childhood Experience (ACEs) and Epigenetic Aging During Pregnancy eTable 4. Association Between Maternal Total Adverse Childhood Experiences (ACE) Score and Newborn Epigenetic Gestational Aging eTable 5. Association Between Individual Maternal Adverse Childhood Experiences (ACEs) and Newborn Epigenetic Gestational Aging eTable 6. Association Between Maternal Depression and Epigenetic Aging During Pregnancy eTable 7. Association Between Maternal Depression During Pregnancy and Newborn Epigenetic Gestational Aging eTable 8. Proportion Mediated by Maternal Depression During Pregnancy for the Association Between Maternal Total Adverse Childhood Experience (ACE) Score and Epigenetic Aging in Mothers and Newborns [file jamanetwopen-e2427063-s001.pdf]

Supplementary Online Content

Dye CK, Alschuler DM, Wu H, et al. Maternal adverse childhood experiences and biological aging during pregnancy and in newborns. *JAMA Netw Open*. 2024;7(8):e2427063. doi:10.1001/jamanetworkopen.2024.27063

eFigure 1. Flowchart for Inclusion Criteria for Study

eTable 1. Epigenetic Clock Statistics From Mothers and Newborns in ALSPAC

eFigure 2. Correlation Matrix of Estimated Epigenetic Age and Chronological Age

eTable 2. Association Between Maternal Epigenetic Aging During Pregnancy and Total Adverse Childhood Experience Score

eTable 3. Association Between Individual Maternal Adverse Childhood Experience (ACEs) and Epigenetic Aging During Pregnancy

eTable 4. Association Between Maternal Total Adverse Childhood Experiences (ACE) Score and Newborn Epigenetic Gestational Aging

eTable 5. Association Between Individual Maternal Adverse Childhood Experiences (ACEs) and Newborn Epigenetic Gestational Aging

eTable 6. Association Between Maternal Depression and Epigenetic Aging During Pregnancy

eTable 7. Association Between Maternal Depression During Pregnancy and Newborn Epigenetic Gestational Aging

eTable 8. Proportion Mediated by Maternal Depression During Pregnancy for the Association Between Maternal Total Adverse Childhood Experience (ACE) Score and Epigenetic Aging in Mothers and Newborns

This supplementary material has been provided by the authors to give readers additional information about their work.

© 2024 Dye CK et al. *JAMA Network Open.*

eFigure 1. Flowchart for Inclusion Criteria for Study

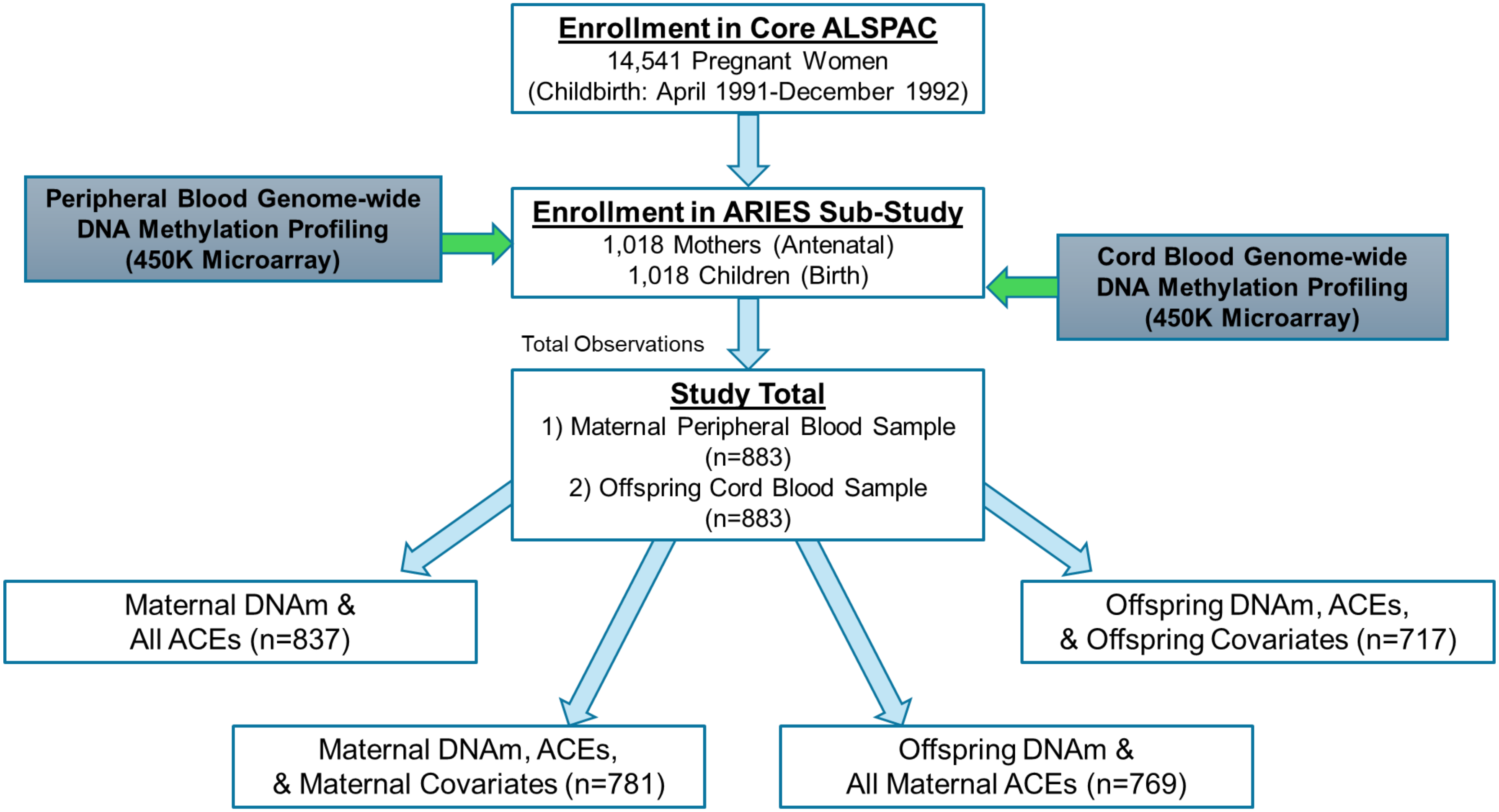

Schematic representation of the ALSPAC mothers during pregnancy (n=14,541) that were enrolled in the ARIES sub-study. In total, 1,018 mother-child dyads were profiled for DNA methylation (DNAm) using the Illumina Infinium HumanMethylation450 BeadChip (450K) to interrogate genome-wide DNAm using peripheral blood taken from peripheral blood from mothers (antenatal) and from cord blood in offspring (birth). Of the 1,018 mother-child dyads, 785 mothers and 753 newborns were retained based on mothers missing less than 3 ACE scores, having high-quality DNAm data collected from peripheral blood (mothers) and cord blood (offspring).

© 2024 Dye CK et al. *JAMA Network Open.*

eTable 1. Epigenetic Clock Statistics From Mothers and Newborns in ALSPAC

Variables	Mean (SD)	Min.-Max.
Maternal Age Characteristics		
Age at delivery (years)	29.75 (4.28)	16.00-42.00
Newborn Age Characteristics		
Gestational age (weeks)	39.59 (1.50)	30.00-40.00
Maternal Epigenetic Age Metrics (years)		
Horvath	39.70 (5.55)	24.60 - 58.66
Hannum	38.28 (4.88)	17.37 - 56.55
PhenoAge	36.57 (6.47)	10.50 - 55.44
GrimAge	48.78 (4.20)	33.35 - 64.33
Maternal Epigenetic Aging (years)		
Horvath	0.00 (4.40)	-12.58 - 18.83
Hannum	0.00 (3.56)	-17.22 - 11.02
PhenoAge	0.00 (5.11)	-21.70 – 15.57
GrimAge	0.00 (2.88)	-12.06 – 13.93
DunedinPACE	1.11 (0.13)	0.63 - 1.56
Newborn Epigenetic Gestational Age Metrics (weeks)		
Knight	38.83 (2.02)	23.90-44.27
Bohlin	39.71 (1.02)	35.24-42.18
Newborn Gestational Aging (weeks)		
Knight	0.00 (1.89)	-14.65 - 5.71
Bohlin	0.00 (0.76)	-3.61 – 2.21

Blood-based maternal epigenetic age metrics are named after Principal Components of respective epigenetic clocks. Epigenetic age changes (i.e., epigenetic age acceleration or deceleration) are based on the residuals between epigenetic age and chronological age. DunedinPACE calculates pace of aging.

© 2024 Dye CK et al. *JAMA Network Open.*

Cord blood-based newborn epigenetic age metrics are named after their respective epigenetic clocks. Biological and gestational aging are based on residuals derived from maternal epigenetic age vs chronological age, and newborn epigenetic gestational age and gestational age, respectively.

Abbrv.: SD: standard deviation; PACE: pace of aging.

© 2024 Dye CK et al. *JAMA Network Open.*

eFigure 2. Correlation Matrix of Estimated Epigenetic Age and Chronological Age

A

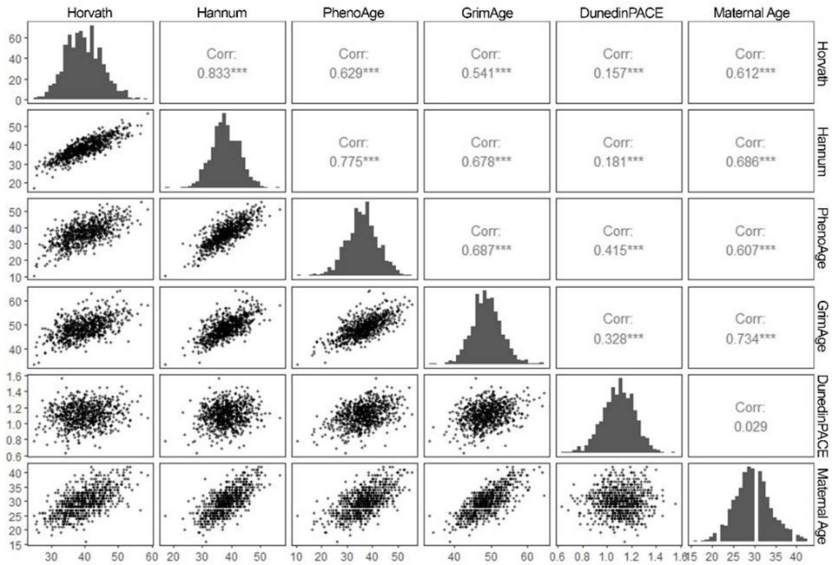

B

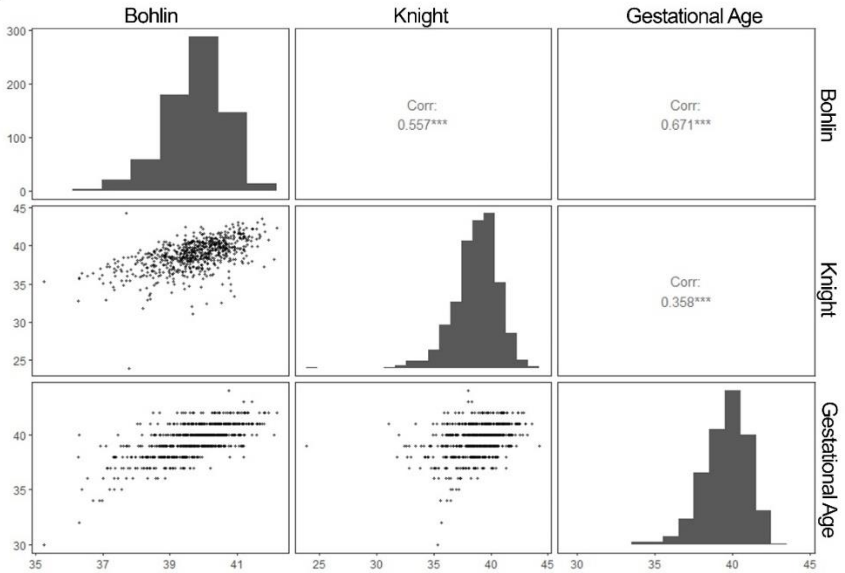

A. Matrix represents the correlation between maternal chronological age (years) and epigenetic age (years) using epigenetic clocks for: Horvath, Hannum, PhenoAge, and GrimAge, and the pace of aging biomarker, DunedinPACE. **B.** Matrix represents the correlation between newborn gestational age (weeks) and epigenetic gestational age estimated using the Bohlin and Knight gestational epigenetic clocks. Significance taken at $p<0.05$; represented by * for <0.05, ** for <0.01, and *** for <0.001.

© 2024 Dye CK et al. *JAMA Network Open.*

eTable 2. Association Between Maternal Epigenetic Aging During Pregnancy and Total Adverse Childhood Experience Score

Epigenetic Clock	Unadjusted			Secondary Model		
	β-Coefficient	95% CI	*p*-value	β-Coefficient	95% CI	*p*-value
Horvath	0.10	-0.10, 0.31	0.32	0.11	-0.10, 0.31	0.31
Hannum	0.05	-0.12, 0.21	0.59	0.06	-0.11, 0.22	0.52
PhenoAge	0.20	-0.04, 0.44	0.10	0.22	-0.02, 0.46	0.08
GrimAge	**0.31**	**0.18, 0.45**	**<0.001**	**0.22**	**0.10, 0.34**	**<0.001**
DunedinPACE	0.00	-0.00, 0.01	0.13	0.00	-0.00, 0.01	0.25

Unadjusted model: minimally adjusted for maternal age.
Secondary Model represents results for the sensitivity analysis for cell type composition.
Secondary Model adjusted for: maternal sample type (white blood cell, whole blood) + maternal age during pregnancy + parity + number of cigarettes smoked during first trimester + maternal education + prenatal BMI (kg/m^2).

© 2024 Dye CK et al. *JAMA Network Open.*

eTable 3. Association Between Individual Maternal Adverse Childhood Experiences (ACEs) and Epigenetic Aging During Pregnancy

	Horvath			Hannum			PhenoAge			GrimAge			DunedinPACE		
ACE Variable	β	(95% CI	*p*-value	β	95% CI	*p*-value	β	95% CI	*p*-value	β	95% CI	*p*-value	β	95% CI	*p*-value
**Primary Model**															
Abuse															
Any type of abuse	0.03	-0.52, 0.58	0.91	0.19	-0.25, 0.63	0.39	**0.79**	**0.22, 1.35**	**0.01**	**0.60**	**0.27, 0.94**	**<0.001**	0.01	-0.01, 0.02	0.38
Emotional	-0.64	-1.58, 0.31	0.19	-0.40	-1.16, 0.36	0.30	0.18	-0.80, 1.16	0.72	**0.75**	**0.18, 1.33**	**0.01**	0.02	-0.01, 0.05	0.26
Physical	0.86	-0.55, 2.28	0.23	0.86	-0.27, 1.99	0.12	1.10	-0.35, 2.56	0.14	0.40	-0.47, 1.26	0.37	0.04	-0.00, 0.08	0.08
Sexual	0.35	-0.23, 0.92	0.24	0.41	-0.05, 0.86	0.08	**0.89**	**0.30, 1.48**	**0.01**	**0.18**	**0.10, 0.79**	**0.01**	0.00	-0.02, 0.02	0.82
Neglect															
Any type of neglect	0.16	-0.50, 0.81	0.63	0.04	-0.48, 0.56	0.88	0.52	-0.15, 1.20	0.13	**0.49**	**0.09, 0.89**	**0.02**	0.00	-0.02, 0.02	0.86
Emotional	0.16	-0.49, 0.82	0.63	0.06	-0.46, 0.59	0.82	0.49	-0.18, 1.17	0.15	**0.52**	**0.12, 0.92**	**0.01**	0.00	-0.02, 0.02	0.94
Physical	-0.35	-2.35, 1.65	0.73	0.22	-1.38, 1.82	0.79	0.02	-2.04, 2.08	0.99	**1.53**	**0.31, 2.74**	**0.01**	0.05	-0.02, 0.11	0.15
Family Adversity															
Any type of family adversity	-0.01	-0.53, 0.51	0.97	0.13	-0.28, 0.55	0.53	0.36	-0.18, 0.90	0.19	**0.46**	**0.14, 0.77**	**0.01**	0.00	-0.01, 0.02	0.63
Parental separation/death	0.35	-0.30, 1.00	0.29	0.10	-0.42, 0.62	0.70	0.02	-0.65, 0.69	0.95	**0.40**	**0.00, 0.80**	**0.05**	0.01	-0.01, 0.03	0.27
Parental domestic violence	-0.32	-1.17, 0.53	0.46	-0.41	-1.09, 0.27	0.24	-0.06	-0.94, 0.82	0.90	**0.62**	**0.10, 1.13**	**0.02**	**0.03**	**0.01, 0.06**	**0.02**
Parental addiction	0.40	-0.56, 1.35	0.41	0.07	-0.69, 0.84	0.85	0.05	-0.94, 1.03	0.93	**0.86**	**0.28, 1.44**	**0.01**	-0.01	-0.04, 0.02	0.53
Parental mental illness	-0.24	-0.83, 0.36	0.44	0.12	-0.36, 0.59	0.63	0.51	-0.10, 1.12	0.10	0.33	-0.04, 0.69	0.08	0.00	-0.02, 0.02	0.86
Parental incarceration	-	-	-	-	-	-	-	-	-	-	-	-	-	-	-
**Secondary**															
Abuse															
Any type of abuse	0.13	-0.54, 0.80	0.70	0.20	-0.34, 0.74	0.47	**0.85**	**0.07, 1.63**	**0.03**	**0.60**	**0.20, 0.99**	**0.00**	0.01	-0.01, 0.03	0.39
Emotional	-0.64	-1.58, 0.31	0.19	-0.40	-1.16, 0.36	0.30	0.18	-0.80, 1.16	0.72	**0.75**	**0.18, 1.33**	**0.01**	0.02	-0.01, 0.05	0.26
Physical	1.07	-0.35, 3.08	0.14	1.13	-0.27, 2.52	0.11	**2.02**	**0.01, 4.03**	**0.05**	0.66	-0.35, 1.68	0.20	**0.05**	**0.00, 0.11**	**0.04**
Sexual	0.34	-0.36, 1.04	0.34	0.30	-0.27, 0.87	0.30	0.81	-0.01, 1.62	0.054	0.36	-0.05, 0.78	0.09	0.00	-0.02, 0.02	0.88
Neglect															

© 2024 Dye CK et al. *JAMA Network Open.*

Any type of neglect	0.62	-0.18, 1.41	0.13	0.15	-0.50, 0.79	0.65	0.58	-0.35, 1.51	0.22	0.46	-0.01, 0.93	0.06	0.00	-0.02, 0.03	0.82
Emotional	0.61	-0.19, 1.41	0.13	0.16	-0.49, 0.80	0.64	0.52	-0.42, 1.45	0.28	**0.48**	**0.00, 0.95**	**0.05**	0.00	-0.02, 0.03	0.91
Physical	0.53	-1.90, 2.96	0.67	0.78	-1.20, 2.75	0.44	1.09	-1.76, 3.94	0.45	**1.81**	**0.37, 3.25**	**0.01**	0.06	-0.01, 0.14	0.08
Family Adversity															
Any type of family adversity	0.04	-0.59, 0.68	0.90	0.06	-0.45, 0.58	0.81	0.31	-0.44, 1.05	0.42	**0.39**	**0.02, 0.77**	**0.04**	0.00	-0.02, 0.02	0.97
Parental separation/death	0.20	-0.59, 1.00	0.62	0.33	-0.64, 0.65	1.00	-0.11	-1.04, 0.82	0.82	0.37	-0.10, 0.84	0.13	0.01	-0.02, 0.03	0.48
Parental domestic violence	-0.17	-1.21, 0.86	0.74	-0.45	-1.28, 0.39	0.30	-0.18	-1.39, 1.03	0.77	0.46	-0.15, 1.07	0.14	0.02	-0.01, 0.06	0.11
Parental addiction	0.42	-0.74, 1.59	0.48	0.20	-0.74, 1.15	0.68	0.47	-0.89, 1.84	0.50	**0.94**	**0.26, 1.63**	**0.01**	0.00	-0.04, 0.03	0.78
Parental mental illness	-0.01	-0.73, 0.72	0.98	0.19	-0.40, 0.78	0.52	0.73	-0.12, 1.58	0.09	0.35	-0.07, 0.78	0.11	0.00	-0.02, 0.02	0.95
Parental incarceration	-	-	-	-	-	-	-	-	-	-	-	-	-	-	-

Linear regression analyses performed between pro-rated individual maternal ACEs and epigenetic age changes calculated using each epigenetic clock, and DunedinPACE.

Primary Model adjusted for: maternal sample type (white blood cell, whole blood) + maternal age during pregnancy + parity + number of cigarettes smoked during first trimester + maternal education + prenatal BMI (kg/m^2) + maternal sample cell type composition (CD4+ T cell, CD8+ T cell, NK cell, monocyte, B cell, neutrophil).
Secondary model is adjusted for: maternal sample type (white blood cell, whole blood) + maternal age during pregnancy + parity + number of cigarettes smoked during first trimester + maternal education + prenatal BMI (kg/m^2).

"Any type" of ACE is considered as having exposure to at least one ACE from category, irrespective of type.
Parental incarceration linear models null due to minimal (n=2 participants) pro-rated values >0.
NA represents not applicable.

© 2024 Dye CK et al. *JAMA Network Open.*

eTable 4. Association Between Maternal Total Adverse Childhood Experiences (ACE) Score and Newborn Epigenetic Gestational Aging

	Unadjusted			Secondary Model		
Epigenetic Clock	β-Coefficient	95% CI	*p*-value	β-Coefficient	95% CI	*p*-value
Female						
Bohlin	0.02	-0.05, 0.09	0.60	0.02	-0.03, 0.07	0.42
Knight	-0.05	-0.18, 0.07	0.40	-0.06	-0.16, 0.05	0.26
Male						
Bohlin	**0.09**	**0.01, 0.17**	**0.04**	0.04	-0.01, 0.10	0.11
Knight	**0.16**	**0.01, 0.31**	**0.04**	0.07	-0.07, 0.20	0.33

Secondary Model represents results for the sensitivity analysis for newborn sample cell type composition.
Secondary Model adjusted for: maternal age during pregnancy + parity + number of cigarettes smoked during first trimester + maternal education + prenatal BMI (kg/m^2) + newborn gestational age + newborn sample type (white blood cells, blood spots) + newborn sample cell type composition (CD4+ T cell, CD8+ T cell, NK cell, monocyte, B cell, neutrophil).

© 2024 Dye CK et al. *JAMA Network Open.*

eTable 5. Association Between Individual Maternal Adverse Childhood Experiences (ACEs) and Newborn Epigenetic Gestational Aging

	Primary Model						Secondary Model					
	Female			Male			Female			Male		
Maternal ACE Variable	β	95% CI	*p*-value	β	95% CI	*p*-value	β	95% CI	*p*-value	β	95% CI	*p*-value
Any type of abuse												
Bohlin clock	0.07	-0.10, 0.24	0.42	0.13	-0.04, 0.30	0.14	0.05	-0.12, 0.21	0.58	0.13	-0.04, 0.30	0.12
Knight clock	-0.03	-0.40, 0.35	0.90	0.25	-0.19, 0.70	0.26	-0.08	-0.42, 0.27	0.67	0.22	-0.20, 0.63	0.31
Any type of neglect												
Bohlin clock	0.15	-0.05, 0.36	0.15	0.14	-0.07, 0.34	0.19	0.12	-0.08, 0.32	0.22	0.09	-0.11, 0.29	0.39
Knight clock	0.15	-0.31, 0.61	0.53	**0.58**	**0.05, 1.11**	**0.03**	0.06	-0.36, 0.48	0.78	0.40	-0.10, 0.90	0.12
Any type of family adversity												
Bohlin clock	0.04	-0.12, 0.21	0.63	0.11	-0.05, 0.28	0.17	0.06	-0.10, 0.22	0.44	0.09	-0.07, 0.25	0.25
Knight clock	-0.25	-0.62, 0.12	0.18	0.06	-0.37, 0.48	0.79	-0.22	-0.55, 0.12	0.21	0.01	-0.39, 0.41	0.98
Emotional Abuse												
Bohlin clock	0.12	-0.16, 0.39	0.41	0.03	-0.28, 0.34	0.86	0.16	-0.11, 0.42	0.25	0.03	-0.27, 0.32	0.86
Knight clock	-0.23	-0.85, 0.38	0.46	0.37	-0.44, 1.18	0.37	-0.10	-0.67, 0.47	0.73	0.33	-0.41, 1.07	0.38
Physical Abuse												
Bohlin clock	0.02	-0.37, 0.41	0.93	0.14	-0.31, 0.58	0.55	0.03	-0.35, 0.41	0.88	0.07	-0.37, 0.50	0.76
Knight clock	-0.04	-0.91, 0.83	0.93	0.45	-0.70, 1.61	0.44	0.06	-0.74, 0.86	0.89	0.30	-0.79, 1.39	0.59
Sexual Abuse												
Bohlin clock	0.06	-0.12, 0.24	0.52	0.12	-0.06, 0.30	0.19	0.04	-0.13, 0.21	0.64	0.09	-0.04, 0.31	0.13
Knight clock	0.04	-0.36, 0.43	0.85	0.13	-0.34, 0.60	0.59	-0.01	-0.37, 0.36	0.98	0.22	-0.32, 0.56	0.59
Emotional Neglect												
Bohlin clock	0.15	-0.05, 0.36	0.15	0.16	-0.04, 0.37	0.12	0.12	-0.08, 0.32	0.22	0.12	-0.09, 0.32	0.26
Knight clock	0.15	-0.31, 0.61	0.53	**0.62**	**0.09, 1.15**	**0.02**	0.06	-0.36, 0.48	0.78	0.45	-0.05, 0.95	0.08
Physical Neglect												
Bohlin clock	-0.19	-0.89, 0.50	0.59	0.00	-0.53, 0.54	0.99	-0.14	-0.81, 0.53	0.68	-0.04	-0.56, 0.48	0.88

© 2024 Dye CK et al. *JAMA Network Open.*

Knight clock	-0.76	-2.31, 0.79	0.34	0.51	-0.89, 1.91	0.47	-0.66	-2.07, 0.76	0.36	0.33	-0.98, 1.63	0.62
Parental separation/death												
Bohlin clock	-0.07	-0.28, 0.13	0.48	0.03	-0.17, 0.23	0.78	0.00	-0.20, 0.19	0.97	0.01	-0.19, 0.20	0.95
Knight clock	**-0.45**	**-0.91, 0.00**	**0.05**	0.49	-0.02, 1.00	0.06	-0.24	-0.65, 0.18	0.27	0.41	-0.07, 0.90	0.10
Parental domestic violence												
Bohlin clock	-0.21	-0.47, 0.05	0.11	0.14	-0.13, 0.42	0.30	-0.21	-0.47, 0.04	0.10	0.11	-0.15, 0.38	0.40
Knight clock	**-0.67**	**-1.25, -0.08**	**0.03**	-0.16	-0.87, 0.55	0.65	**-0.70**	**-1.23, -0.16**	**0.01**	-0.25	-0.92, 0.42	0.46
Parental addiction												
Bohlin clock	0.24	-0.06, 0.54	0.11	0.34	0.03, 0.65	0.03	0.21	-0.08, 0.50	0.16	0.28	-0.02, 0.58	0.07
Knight clock	-0.15	-0.83, 0.52	0.65	0.49	-0.32, 1.30	0.24	-0.27	-0.89, 0.34	0.38	0.21	-0.56, 0.97	0.59
Parental mental illness												
Bohlin clock	0.04	-0.14, 0.23	0.65	0.12	-0.06, 0.30	0.19	0.09	-0.09, 0.27	0.35	0.08	-0.10, 0.25	0.40
Knight clock	-0.15	-0.57, 0.26	0.46	-0.12	-0.59, 0.36	0.63	-0.09	-0.47, 0.30	0.66	-0.24	-0.68, 0.20	0.28
Parental incarceration												
Bohlin clock		NA			NA			NA			NA	
Knight clock		NA			NA			NA			NA	

Linear regression analyses performed between pro-rated individual maternal ACEs and epigenetic gestational age changes calculated using each gestational epigenetic clock separately for male and female newborns in regression models.

Primary model adjusted for: maternal age during pregnancy + parity + number of cigarettes smoked during first trimester + maternal education + prenatal BMI (kg/m^2) + newborn gestational age + newborn sample type (white blood cells, blood spots).
Secondary model is adjusted for: maternal age during pregnancy + parity number of cigarettes smoked during first trimester + maternal education + prenatal BMI (kg/m^2) + newborn gestational age + newborn sample type (white blood cells, blood spots) + newborn sample cell type composition (CD4+ T cell, CD8+ T cell, NK cell, monocyte, B cell, neutrophil, nucleated red blood cell).
NA represents not applicable.

© 2024 Dye CK et al. *JAMA Network Open.*

eTable 6. Association Between Maternal Depression and Epigenetic Aging During Pregnancy

	Unadjusted			Primary Model			Secondary Model		
Epigenetic Clock	β-Coefficient	95% CI	*p*-value	β-Coefficient	95% CI	*p*-value	β-Coefficient	95% CI	*p*-value
Horvath	0.05	-0.02, 0.12	0.19	0.03	-0.03, 0.09	0.30	0.06	-0.01, 0.13	0.11
Hannum	0.00	-0.05, 0.06	0.97	0.01	-0.04, 0.07	0.63	0.02	-0.05, 0.08	0.60
PhenoAge	-0.02	-0.10, 0.06	0.68	0.00	-0.07, 0.06	0.91	-0.01	-0.10, 0.07	0.81
GrimAge	0.08	0.03, 0.12	**<0.001**	**0.06**	**0.02, 0.10**	**0.01**	0.05	**0.01, 0.10**	**0.02**
DunedinPACE	0.00	-0.00, 0.00	0.93	0.00	-0.00, 0.00	0.47	0.00	-0.00, 0.00	0.50

Depression measured using the Edinburgh Postnatal Depression Scale using a summary score across 10 questions.

Unadjusted model: minimally adjusted for maternal age.

Primary Model adjusted for: maternal sample type (white blood cell or whole blood) + maternal age during pregnancy + parity + number of cigarettes smoked during first trimester + maternal education + prenatal BMI (kg/m^2) + maternal sample cell type composition (CD4+ T cell, CD8+ T cell, NK cell, monocyte, B cell, neutrophil).

Secondary Model adjusted for: maternal sample type (white blood cell or whole blood) + maternal age during pregnancy + parity + maternal smoking status during pregnancy + maternal education + pre-pregnancy BMI (kg/m^2).

© 2024 Dye CK et al. *JAMA Network Open.*

eTable 7. Association Between Maternal Depression During Pregnancy and Newborn Epigenetic Gestational Aging

	Unadjusted			Primary Model			Secondary Model		
Epigenetic Clock	β-Coefficient	95% CI	*p*-value	β-Coefficient	95% CI	*p*-value	β-Coefficient	95% CI	*p*-value
Female									
Bohlin	0.00	-0.03, 0.02	0.88	0.0002	-0.02, 0.02	0.85	0.00	-0.02, 0.02	0.98
Knight	0.00	-0.05, 0.04	0.86	-0.01	-0.05, 0.03	0.76	0.00	-0.04, 0.04	0.94
Male									
Bohlin	0.01	-0.02, 0.04	0.44	0.01	-0.01, 0.03	0.24	0.01	-0.01, 0.03	0.24
Knight	0.03	-0.02, 0.08	0.25	0.03	-0.02, 0.07	0.24	0.02	-0.02, 0.07	0.28

Primary Model is adjusted for: adjusted for maternal age during pregnancy + parity + number of cigarettes smoked during first trimester + maternal education + prenatal BMI (kg/m^2) + gestational age at birth + newborn sample type (white blood cells, blood spots).

Secondary Model adjusted for: maternal age during pregnancy + parity + maternal smoking status during pregnancy + maternal education + pre-pregnancy BMI (kg/m^2) + newborn gestational age + newborn sample type (white blood cells or blood spots) + newborn sample cell type composition (CD4+ T cell, CD8+ T cell, NK cell, Monocyte, B cell, neutrophil, nucleated red blood cell).

© 2024 Dye CK et al. *JAMA Network Open.*

eTable 8. Proportion Mediated by Maternal Depression During Pregnancy for the Association Between Maternal Total Adverse Childhood Experience (ACE) Score and Epigenetic Aging in Mothers and Newborns

Epigenetic Clock	β-Coefficient	Standard Error	*p*-value
Maternal Model			
Horvath	0.02	0.02	0.24
Hannum	-0.003	0.01	0.83
PhenoAge	-0.02	0.02	0.29
GrimAge	0.02	0.01	0.10
Male Offspring Model			
Bohlin	0.01	0.01	0.25
Knight	0.02	0.02	0.27
Female Offspring Model			
Bohlin	0.00	0.00	0.91
Knight	-0.001	0.01	0.90

Indirect effect is used to describe the effects of the maternal total ACE score on epigenetic aging (i.e., EAA and GAA) in mothers and newborns that is mediated through maternal depression (i.e., EPDS).

Maternal models are adjusted for: maternal sample type (white blood cell, whole blood) + maternal age during pregnancy + parity + number of cigarettes smoked during first trimester + maternal education + prenatal BMI (kg/m2).

Offspring models are adjusted for: maternal age during pregnancy + parity + number of cigarettes smoked during first trimester + maternal education + prenatal BMI (kg/m2) + gestational age at birth + newborn sample type (white blood cells, blood spots).

© 2024 Dye CK et al. *JAMA Network Open.*
