# Supplementary material for: Maternal Adverse Childhood Experiences and Biological Aging During Pregnancy and in Newborns
Source: JAMA Netw Open. 2024 Aug 9;7(8):e2427063. doi: 10.1001/jamanetworkopen.2024.27063 (PMC11316241; doi:10.1001/jamanetworkopen.2024.27063)
Supplement: Supplement 2. — Data Sharing Statement [file jamanetwopen-e2427063-s002.pdf]

Data Sharing Statement

Dye. Maternal Adverse Childhood Experiences and Biological Aging During Pregnancy and in Newborns. *JAMA Netw Open*. Published August 09, 2024.
doi:10.1001/jamanetworkopen.2024.27063

Data
Data available: Yes
Data types: Data dictionary
How to access data: http://www.bris.ac.uk/alspac/researchers/data-access/data-dictionary/
When available: With publication

Supporting Documents
Document types: None

Additional Information
Who can access the data: Data available from ALSPAC and the ARIES sub-study are listed at http://www.bristol.ac.uk/alspac/ , which contained details of all data available through a fully searchable data dictionary. Access to existing data, including those used in this study, will be granted upon approval by the ALSPAC Executive committee.
Types of analyses: Data will be made available for all analyses outlined in a formal proposal submitted and approved by the ALSPAC Executive Committee, and will not be made available otherwise.
Mechanisms of data availability: Data will be made available only after approval of a proposal by the ALSPAC Executive Committee.
